# Supplementary material for: Conceptual qualitative system dynamics model for simulation of perceived workload, stress and performance from industrial work content
Source: PLoS One. 2026 May 4;21(5):e0347030. doi: 10.1371/journal.pone.0347030 (PMC13138633; doi:10.1371/journal.pone.0347030)
Supplement: S1 Table — Terms that are used in the proposed model are explained in the Table below. The terms are sorted in the order of appearance throughout the work. (PDF) [file pone.0347030.s001.pdf]

S1 Table. The glossary of relevant terms: Terms that are used in the proposed model are explained in the Table below. The terms are sorted in the order of appearance throughout the work.

Norm	Explanation and Scope
primary stressor	The demand of the task as the main stressor that imposes stress on the workers, including mental and physical ”task load”
task load	The demand of the work task, including components such as posture, force, and time in physical ”task load”, and visual, auditory, cognitive, and psychomotor in mental ”task load”
perceived workload	The perceived value of task load, with components corresponding to those of task load, that depend on the ”personal perception” of each worker.
workload component interaction	the phenomenon when the components of ”task load” create additional value in workload perception
interacted load	The additional value occurs due to ”workload component interaction”
workload	the total value of load that is perceived by the workers, which is the sum of ”perceived workload” and ”interacted load”
personal perception	The mechanism of how an individual worker experiences the ”task load”, and forms the ”workload”
personal profile	The factors that define the background information of an individual worker, from professional and occupational aspects, divided into ”static profile”, ”dynamic profile”, and ”stress-related profile
static profile	The factors of ”personal profile” that are mostly static and undergo a long process of change, such as age, work experience
dynamic profile	The factors of ”personal profile” that are dynamic and can be changed within a short time period, such as training experience, skill decay
stress-related profile	The factors of ”personal profile” that are related to the stress accumulation mechanism of a person, such as stress endurance
work capacity	The limited capacity for work in percentage, with six components corresponding to ”task load”, namely posture, force, visual, auditory, cognitive, and psychomotor, except for the ”time” load
initial personal capacity	The ”work capacity” of an individual worker, which is dependent on the ”personal profile”
personal capacity	The ”work capacity” of an individual worker at a certain time of simulation
natural degradation	The natural rate at which the ”personal capacity” degrades, under normal working conditions and with ”basic task load”
stress degradation	The rate at which the ”personal capacity” degrades when the worker experiences stress
capacity degradation	The total rate of ”personal capacity” degradation at a certain time, equals the sum of ”natural degradation” and ”stress degradation”
motivated capacity	The increment in ”personal capacity” due to the external motivation
time variation	The variation in cycle time of a certain task conducted by an individual worker
time pressure	The perceived stress induced by time
basic task load	The basic demand of each ”task load” component, that an individual can work throughout the shift without experiencing fatigue
basic time load	The basic demand of ”task load” in terms of time, including ”basic task time” and ”basic pace time”
basic task time	The allowed time to perform a certain task
basic pace time	The allowed time between incoming tasks, or cycle time between work cycles
secondary stressor	The factors of the working environment, which are different from ”task load”, act as the secondary stressors that impose stress on the workers, including static and dynamic factors and yielding static and dynamic effects
workload reception	The process in which the worker receives the ”task load” and forms ”workload”
perceived situational demand	The level of demand that an individual worker feels from the difference between ”task load” and ”basic task load”
perceived capability	The level of workability that an individual worker feels from the difference between current ”capacity degradation” and the ”natural degradation”
sustained attention	The status in which the worker is constantly focusing on the current task, with values that can be accumulated and reduced within a work shift
acute stress	The short-term status in which the worker feels stressed, with values that can be accumulated and reduced within a work shift
chronic stress	The long-term status in which the worker feels stressed, with values that (in this study) can only be accumulated within a work shift
accumulation rate	The accumulation in value of different stress types
relaxation rate	The reduction in value of different stress types
under-load	The status in which the worker does not experience any stress, due to a low value of ”task load”
optimal performance	The status in which the worker experiences an acceptable ”workload”, with aroused vigilance and task engagement
overload	The status in which the worker experiences an excessive level of ”workload”
performance profile	The probabilities that the worker can fulfill the requirement in ”availability”, ”productivity”, and ”quality” aspects at a certain time during work shift
intervention	The changes or modifications in ”task load”, or managerial activities, that interfere with the work process
