# Supplementary material for: Conceptual qualitative system dynamics model for simulation of perceived workload, stress and performance from industrial work content
Source: PLoS One. 2026 May 4;21(5):e0347030. doi: 10.1371/journal.pone.0347030 (PMC13138633; doi:10.1371/journal.pone.0347030)
Supplement: S2 Table — (PDF) [file pone.0347030.s002.pdf]

S2 Table. The proposed scopes of physical and mental "task load" in the proposed model are discussed in the Table below.

Type	Measured in relevant study	Component	Scope	Direction of effect
Physical workload	HR [29, 131, 250], energy expenditure [29], RPE [29, 130], calorie consumption [170], oxygen uptake [131], blood pressure, gas exchange [130].	Posture	Posture required to perform the task	Posture "task load" (non-ergonomic, leg imbalance, with out-range movements) causes posture "workload" (e.g., pain [96]) and cognitive "workload" (e.g., tiredness [90]).
		Force	The force that should be exerted.	High force "task load" leads to force "workload" (e.g., muscle fatigue [98]) and psychomotor "workload" (e.g., degrades physical force capacity [97]).
		Time	The cycle time for a task, and the work pace between tasks.	Time "task load" (i.e., short cycle time or fast work pace [99]) induces cognitive "workload" (i.e., stress) and force "workload" (i.e., sustained force) [133]. Time and posture "task load" (e.g., repetitive motions [100]) causes posture "workload" (e.g., musculoskeletal disorders [97, 101]). Reasonable time "task load" (e.g., flexible work/rest ratio [175]) induces positive time and cognitive "workload" (e.g., allows more control and stress relaxation).
Mental workload	Subjective measures: NASA-TLX [170, 250], MRQ, JCQ [201], VACP [251]. Physiological signals: ECG, EMG, EEG, eye movement, HR, HRV, respiration, etc. [252].	Visual	Visual activities and efforts.	Demanding visual "task load" [102] increases the visual "workload" and degrades the task performance.
		Auditory	Auditory activities and efforts.	High listening "task load" can lead to force "workload" (e.g., fatigue [253]), thus decreasing hearing abilities and performance [254].
		Cognitive	Cognitive activities and efforts.	High cognitive "task load" results in greater force and cognitive "workload" (e.g., muscle activity [103] and decreased motivation [255]), decreasing finish time and performance [104].
		Psycho-motor	Psychomotor activities and efforts.	Intensive psychomotor "task load" places an additional cognitive "workload" (e.g., burden and mental engagement [256]).

ECG: Electroencephalography, EMG: Electromyogram, EEG: Electroencephalogram. JCQ: Job Content Questionnaire
HR: Heart rate. HRV: Heart rate variability. MRQ: Multiple Resource Questionnaire. RPE: Rating of Perceived Exertion.
VACP: Visual, Auditory, Cognitive, Psychomotor.
