# Supplementary material for: Conceptual qualitative system dynamics model for simulation of perceived workload, stress and performance from industrial work content
Source: PLoS One. 2026 May 4;21(5):e0347030. doi: 10.1371/journal.pone.0347030 (PMC13138633; doi:10.1371/journal.pone.0347030)
Supplement: S3 Table — (PDF) [file pone.0347030.s003.pdf]

S3 Table. Different factors considered in a personal profile during the model development are described in Table below.

Type	Factor	Scope	Direction of effect
Static profile	Work experience	The duration of working in a position with similar requirements.	Performance gradually increases with work experience but decreases slightly after a threshold of 20 years due to work boredom [257].
	Age effect	The effect of aging on personal working capacities.	Posture, muscular power, and psychomotor functions decline with age, up to a threshold of 20% year between 40-60 years [258], especially after a threshold of 50 year [259, 260]; with lower sustained attention limit, reduced training and learning efficiency [260], reduced stress resilience and adaptation [261].
	Physical impairment	The impaired health condition reduces work functioning [262].	Workers with physical impairment have reduced work capability and productivity [263], requiring improved resources and support to avoid stress and frustration [264].
	Shift work	The accumulating hours working on the night shift or rotating shift.	Working at night with abnormal working hours [265, 266] plus the risk of sleepiness decreases the psychomotor [267], cognitive [268] and posture capacity [269].
	Sleep quality	The sleep-wake cycle of an undisturbed sleep pattern.	Frequently disrupted and restricted sleep causes disorders that reduce stress endurance [270], thus leading to mental fatigue and burnout [271].
	Chronic stress effect	The accumulated long-term stress from daily life events.	Chronic stress reduces cognitive ability [272], increases vulnerability to mental illness, and decreases the stress recovery ability [273].
	Job motivation	The incentive level to carry out the assigned task in the work position.	Well-motivated workers have better stress endurance to avoid emotional exhaustion [274], and are willing to spend more effort and persistence on their task [275].
Dynamic profile	Training experience	The duration of being trained in the current assigned position.	A sufficient amount of training helps to increase psychomotor fatigue threshold [276] and prevent significant psychomotor performance degradation [277].
	Learning ability	The reduced task time variation and defect rate in repetitive tasks.	The actual cycle time will be reduced after a threshold of X finished products [278] due to familiarity with the operation and tools [279]. The value of X can be predefined according to the task characteristics.
	Skill decay	The task time variation, reflecting the skill proficiency in the current position.	The position-related skills naturally undergo a gradual exponential decay. Regular reviews and refresher training help to maintain the values [126].
	Problem-solving ability	The skills and confidence to solve production problems.	High problem-solving ability increases job control [133], thus reducing the perceived workload from occurring problems and positively impacting the performance [280].
Stress-related profile	Stress endurance	The personal resilience against sustained attention and stressful situations.	Mental toughness as a personality helps the worker in stress coping [281], and becomes stress resilient with a low level of anxiety and enhanced physical endurance [282]. Mindfulness and self-efficacy attenuate stress that impairs performance [283]
	Task demand threshold	The personal limit of workload that is acceptable for the worker.	When the task demand requires higher effort than the worker can dedicate, the perception of workload becomes a negative process with decrements in performance or willingness to perform [284].
	Capability threshold	The personal limit of capability degradation at which the worker does not feel a burden.	Significant physical and mental capacities deterioration that exceeds the "natural degradation" can cause reduced professional efficacy, which refers to feelings of insufficiency, incompetence, under-productiveness [285] and burnout [286]. These threshold values can be adjusted with personalized values.
	Sustained attention threshold	The minimum and maximum value and duration of sustained attention.	Tasks that lack alertness with a low level of sustained attention cause drowsiness [141], while a prolonged duration of vigilance stimulates acute stress [51].
	Acute stress threshold	The maximum value and duration of acute stress before it transforms into chronic effect.	Exposure to a certain level of acute stress results in sustained remodeling of neuroarchitecture, which leaves a long-term disorder outcome lasting for 24 hours or more [287].
