# Supplementary material for: Conceptual qualitative system dynamics model for simulation of perceived workload, stress and performance from industrial work content
Source: PLoS One. 2026 May 4;21(5):e0347030. doi: 10.1371/journal.pone.0347030 (PMC13138633; doi:10.1371/journal.pone.0347030)
Supplement: S4 Table — (PDF) [file pone.0347030.s004.pdf]

S4 Table. Circumstantial stressors and their effects from the literature are described in Table below.

Type	Factor	Scope	Direction of effect
**Work setting: Static**	Environment disturbance	The environmental comfort from physical, functional and psychological aspects [60].	Physical environment can either support the tasks, activities, learning efficiency [288] and the cognitive function [289] with comfort condition, or slows them down with uncomfortable condition and stress [3].
	Work pace	The interval of incoming tasks.	Intensive pace than normal will be demanding and cause stressful perception [133].
	Buffer capacity	The number of WIP in buffer inventory.	No buffer caused reduced line output due to personal task time variation [290], low inventory instills fear of causing idle time, thus the slow worker tends to work faster [169], too high inventory causes waste and idle time. A suitable buffer allows pace control and compensates for task time variation, thus reducing stress [175].
	Pattern change	The changes in the assigned incoming tasks or product sequences, or job rotation during a working session.	The change in work pattern reduces the monotony and ergonomic risk [291, 292] and supports the recovery from demanding tasks and position [177], reduces assembly errors and enhances product quality [293].
	Ergonomic layout	The ergonomic design of the work cell and work layout.	Worker- and work-oriented ergonomics improves performance [294], while ergonomic difficulty hinders work movements, thus induces stress [133].
	Support readiness	Social support from co-workers or advisors.	Support from team and supervisors reducing stress [133, 201], while absentee co-workers increase work pace and intensity, thus increasing physical demand and stress [133].
**Work characteristic: Dynamic**	Weekly working hours	The currently accumulating working hours from the beginning of the week.	Longer weekly working hours than desired increases physical demand [175], decreases all the capabilities, and increases stress during the extra hours [133, 295].
	Failure rate	The occurrence rate of failures or problems during work.	Failures/defects create blame feelings that persist for a long time after occurrence [133], high occurring frequency poses additional pressure [175].
	Body asymmetry	The asymmetric difference of body part utilization.	Asymmetric task design or work behavior reduces work capacity [296], causing muscle fatigue [176] and musculoskeletal pain [297], thus negatively impacting the perceived workload and reducing the recovery rate [176].
	Finished product	The accumulating number of finished products.	The cycle time is shortened due to the learning effect after a certain number of finished products [278]. The visualization of finished individual output facilitates personal commitment, therefore reducing perceived stress [175].
	Problem complexity	The difficulty of the occurring failure/defect/problem.	A complex problem with no previous training poses an extra workload, causing idle time. Problems that lead to line stoppages induce stressful impressions [133, 175].
