# Supplementary figures and images for: Conceptual qualitative system dynamics model for simulation of perceived workload, stress and performance from industrial work content
Source: PLoS One. 2026 May 4;21(5):e0347030. doi: 10.1371/journal.pone.0347030 (PMC13138633; doi:10.1371/journal.pone.0347030)

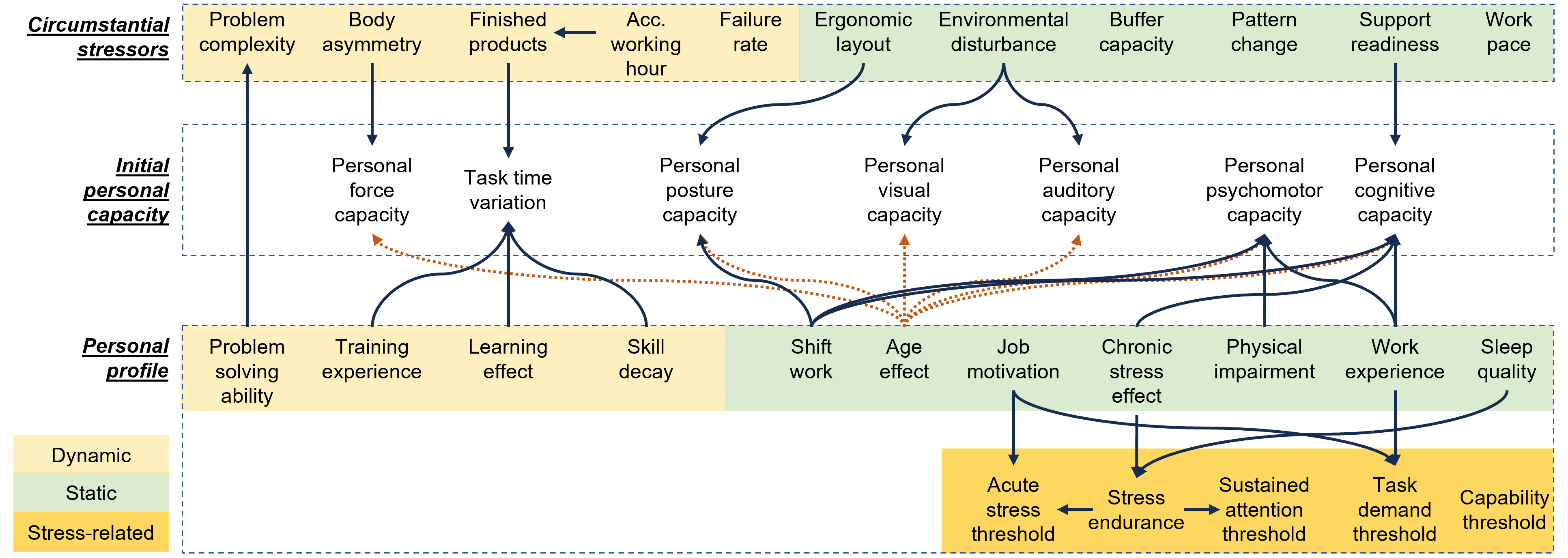

Supplement: S1 Fig — For better comprehension, the model structure in the Vensim environment was developed in separate views, with each view represented in the following figures. (TIF) [file pone.0347030.s005.tif]

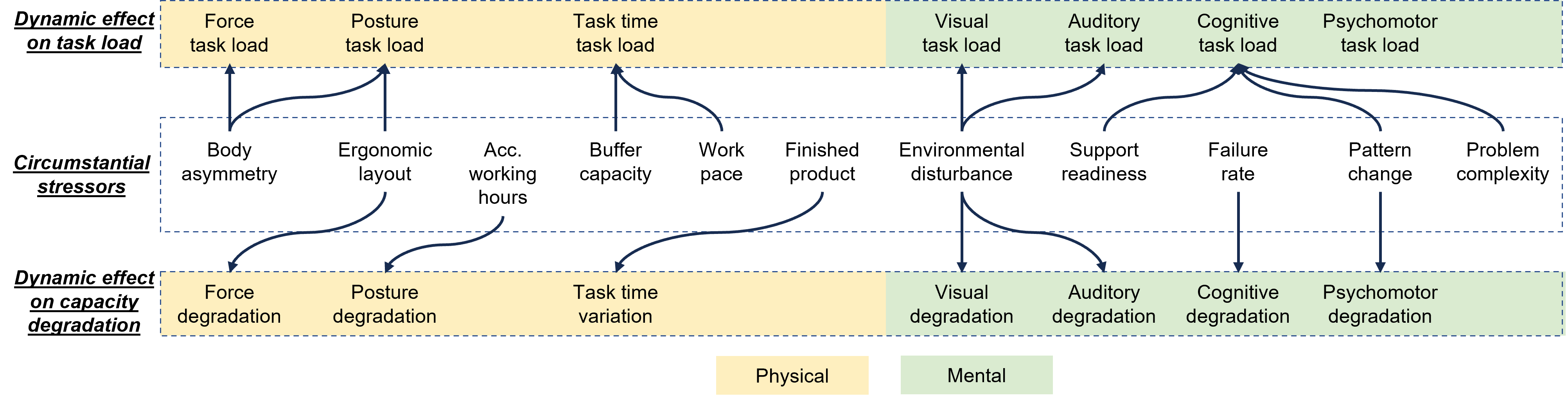

Supplement: S2 Fig — The dynamic effects of circumstantial stressors on the perceived workload and working capacity degradation. (TIF) [file pone.0347030.s006.tif]

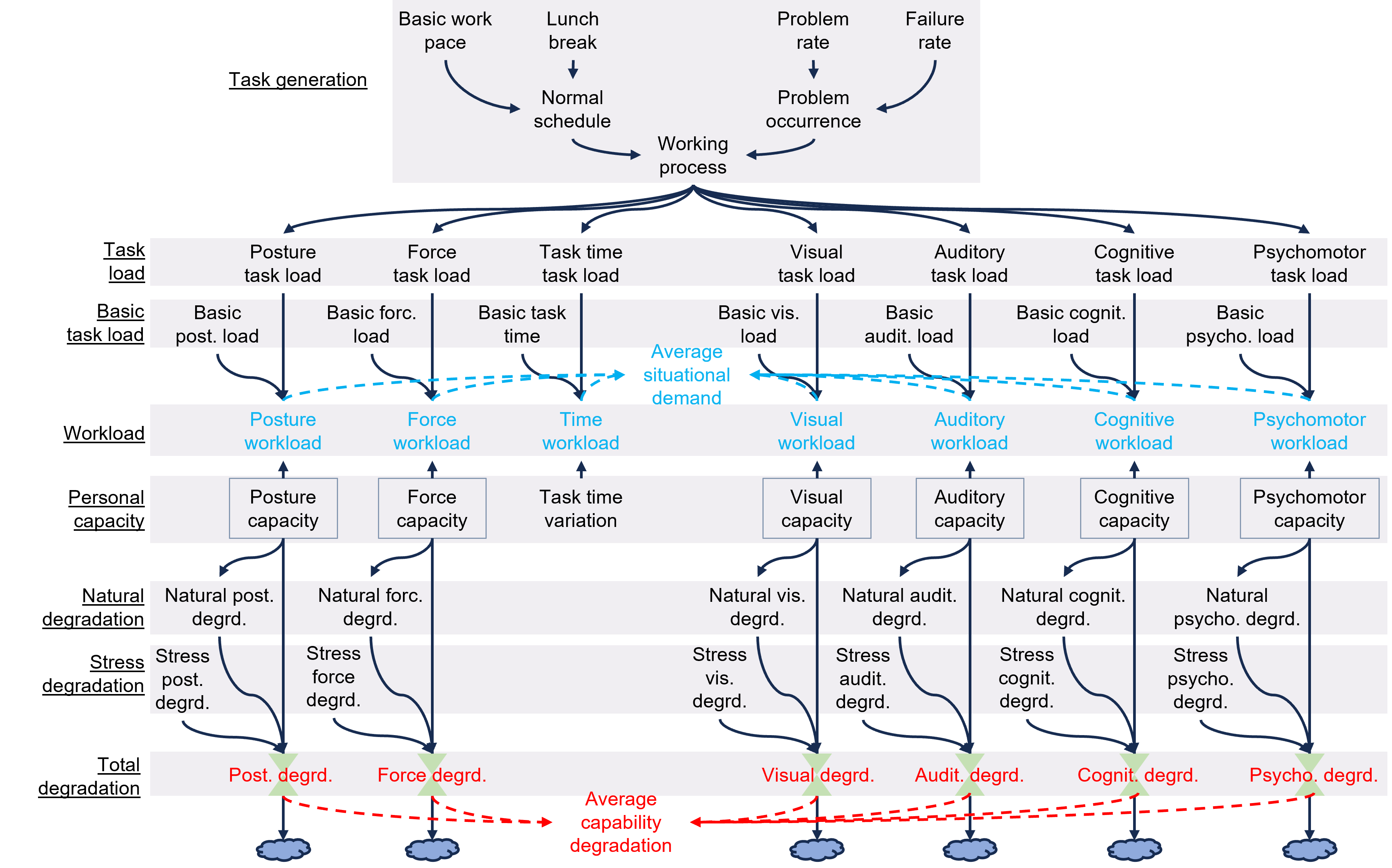

Supplement: S3 Fig — This Fig visualizes the model structure to generate task load components, and how the work capacities are modeled by stock variables. (TIF) [file pone.0347030.s007.tif]

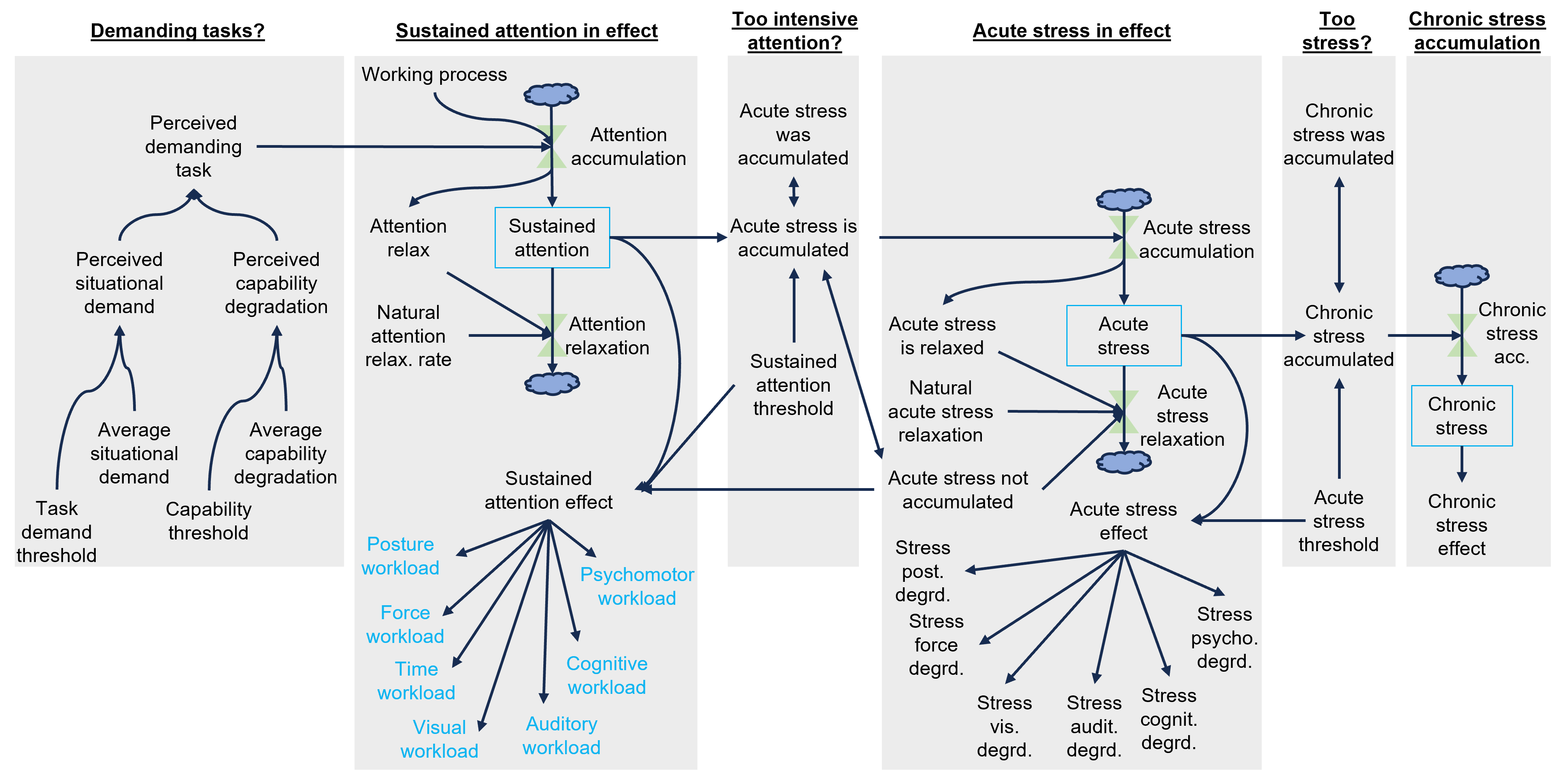

Supplement: S4 Fig — This Fig describes the structure to create the accumulation and relaxation mechanism of different stress types and their effects on workload and capacity degradation. (TIF) [file pone.0347030.s008.tif]

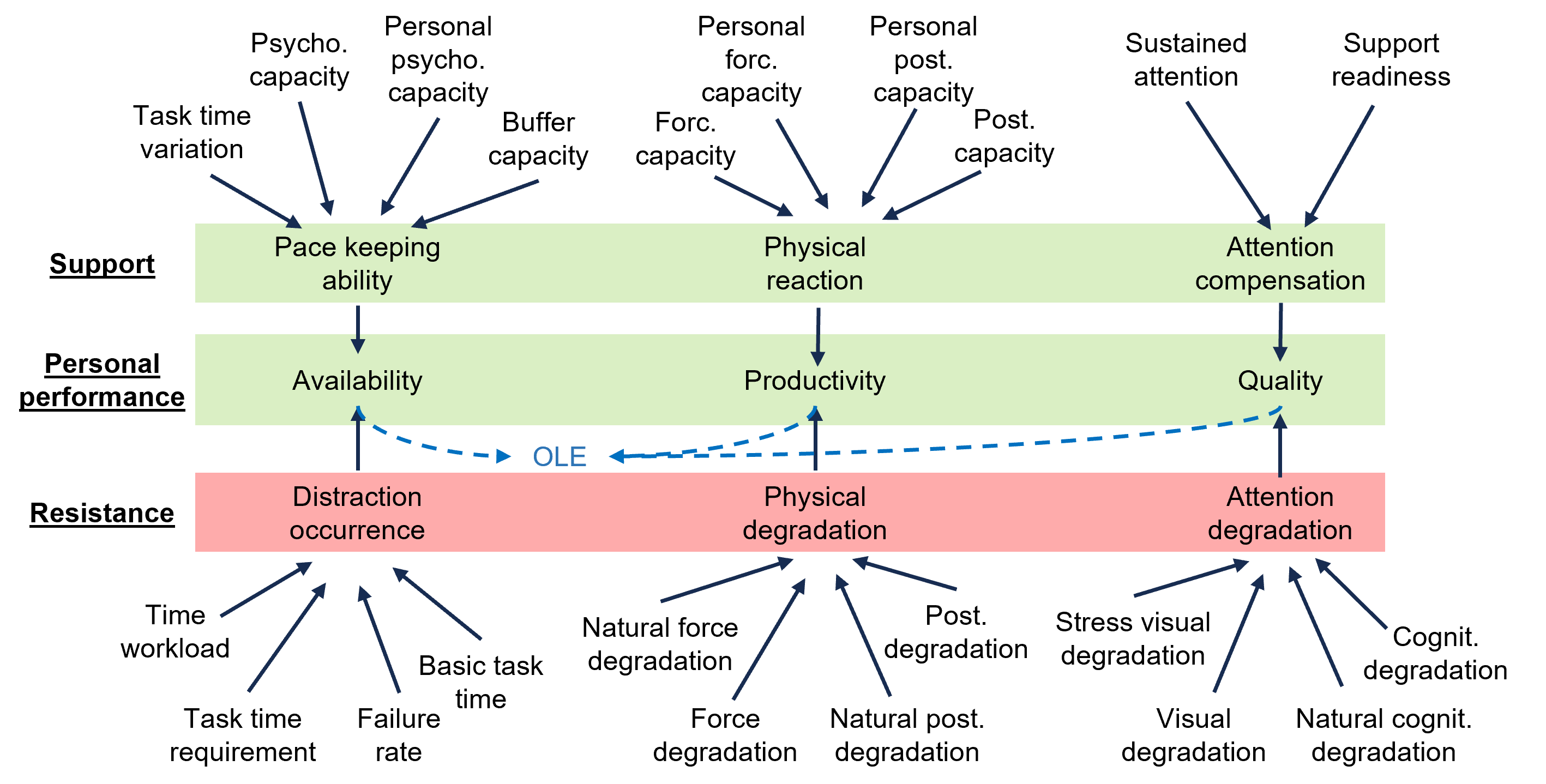

Supplement: S5 Fig — The OLE is defined by its constituents. (TIF) [file pone.0347030.s009.tif]
