# Supplementary material for: Conceptual qualitative system dynamics model for simulation of perceived workload, stress and performance from industrial work content
Source: PLoS One. 2026 May 4;21(5):e0347030. doi: 10.1371/journal.pone.0347030 (PMC13138633; doi:10.1371/journal.pone.0347030)
Supplement: S5 Table — The details of the use case setup with the important variables, along with their values and equations. Relevant experiment data or theoretical models used are mentioned. Given that no study mentioned the exact scope of the use case, only the general shapes of the curve or the function from these studies are incorporated to generate the inputs of the model. Other variables were constructed with simplified relationships from input variables, based on reasoning from relevant theoretical studies. (PDF) [file pone.0347030.s010.pdf]

S5 Table. Model variables, values, and equations. The details of the use case setup with the important variables are described, along with their values and equations. Relevant experiment data or theoretical models used are mentioned. Given that no study mentioned the exact scope of the use case, only the general shapes of the curve or the function from these studies are incorporated to generate the inputs of the model. Other variables were constructed with simplified relationships from input variables, based on reasoning from relevant theoretical studies.

Type	Variable (#)	Explanation/Reasoning	Value/Equation	Relevant experiment data/theoretical model
Personal profile	Work experience (C)	The positive effect of experience on personal initial cognitive and psychomotor capacities, task demand threshold, work performance.	A lookup function by year value from an exponential curve, which has different shapes for individuals.	Meta-analysis (N = 25.911, K = 44) [298], qualitative assessment (N = 176 manufacturing workers) [299]
	Age effect (C)	The negative effect on all personal initial capacity (except time) with an increase in age.	A lookup function by year value from an exponential curve, which is relatively constant until the threshold of 45 and significantly declines afterward.	Cross-sectional study on 701 healthy workers from 180 professions with different physical load [197]
	Physical impairment (C)	The individual physical fitness affects all personal initial capacities and maximum work capability threshold.	A lookup function by individual psychophysical variables.	Experiment with 135 industrial workers and students [300])
	Shift work (C)	Put a zero effect for the day shift and an hourly cumulative negative effect for the night shift [301].	A lookup function by working hours from the shift beginning.	Experiment with 29 mining operators in cognitive testing [302]
	Sleep quality (C)	The negative effect of sleep quality on all personal initial capacities and capability threshold [303]	A lookup function by sleep quality score [304].	Meta-analysis of 68 studies on the effect of sleep quality and quantity on individual health outcomes [303]
	Chronic stress effect (C)	The negative effect on poor concentration, irritability, reduced stress tolerance [305].	A lookup function by allostatic load level (assessed by biochemical marker [306]).	Psychological data analysis of 40 participants under severe occupational stress [307]
	Job motivation (C)	The intrinsic motivation mediates the effect of stressors and improves stress endurance [308].	A lookup function by work motivation score [309].	Online survey from 284 participants on work motivation
	Training experience (C)	Training has a positive effect on all personal initial capacities, in both cognitive and psychomotor areas [310].	A lookup function by the number of finished products/cycles from the learning curve.	Experiment with 37 participants during training phase [311]
	Learning ability (C)	The individual capability of learning rate on personal task time and task time variation [312].	A lookup function of individual learning rate by prior experience.	3874 episodes of individual performance from assembly workers [313]
	Skill decay (A)	The percent of task time variation.	A lookup function by weeks without practice from the skill decaying curve [314].	Follow-up study with 1000 rail safety workers [314]
	Problem-solving ability (C)	The positive effect of individual problem-solving skills on reducing the perceived workload from occurring problems [315].	A lookup function by the personal problem-solving score [316].	Questionnaire about self-efficacy, coping style on stressors from 2293 U.S. army members [315]

Continued on the next page

Type	Variable (#)	Explanation/Reasoning	Value/Equation	Relevant experiment data/theoretical model
	Stress endurance (A)	The personal threshold for different types of stress.	Stress endurance = - Chronic stress effect - Sleep quality - Weekly working hours	-
	Task demand threshold (A)	Personal threshold for perceived situational demand.	Task demand threshold = Basic task load* + Job motivation + Work experience	-
	Capability threshold (A)	Personal threshold for working capacity degradation.	Capability threshold = Average of Natural degradation rates - Physical impairment	-
	Stress threshold (C)	Personal threshold for different types of stress.	Stress threshold = Lookup functions by perceived stress score [317] for each type of stress + Stress endurance.	-
**Circumstantial stressor**	Environmental disturbance (C)	Zero effect if the environment is in normal condition, negative effect with unfavorable conditions.	A lookup function by environmental comfort parameters [318]	Questionnaires about work comfort and productivity of 15 molders [319]
	Work pace (C)	The predefined value for work pace between incoming tasks.	An input constant in minutes.	-
	Buffer capacity (C)	The cycle time variation that is allowed by the number of work-in-process buffers.	A lookup function by buffer quantity.	Laboratory simulation of production line [321]
	Pattern change (C)	The positive effect on reduced cognitive workload and psychomotor degradation.	A lookup function by the boredom cost of employee [322].	Self-report and objective measures of effect from work patterns on stress and fatigue of 177 offshore workers
	Ergonomic layout (C)	The positive effect on reduced posture and psychomotor workloads.	A lookup function by RULA [323] score.	20 recorded trials from 10 laborers in focus group study [324]
	Support readiness (C)	The positive effect of external support on cognitive capacity.	A lookup function by social support score (assessed by JCQ [201])	Questionnaire about the effect of support from coworker and supervisor on the perceived workload of 150 staff [325]
	Weekly working hours (C)	The negative effect of long working hours on mental health, performance, safety [326].	A lookup function by weekly working hours.	Samples of individual full-time and part-time workers from the US General Social Survey during 1972-2012 [327]
	Failure rate (C)	The occurring rate of failure/problem.	An input constant in minutes.	-
	Body asymmetry (C)	The percentage of asymmetry when using body parts.	An input constant in percent.	-
	Finished products (A)	The accumulating number of finished products after the incoming task.	Finished products = Task schedule / (Work pace + Actual task time)	-
	Problem complexity (C)	The difference of additional workload caused by the occurred problem compared to the basic task load.	An input constant in percent, with different values for different complexity levels.	-
**Initial condition**	Initial personal capacity* (A)	The work capacities of each individual (except time) at the beginning of work session, depending of personal profile and the circumstantial stressors in the assigned position.	Initial personal capacity = 100 - Effect from personal profile + Effect from Circumstantial stressor	-

Continued on the next page

Type	Variable (#)	Explanation/Reasoning	Value/Equation	Relevant experiment data/theoretical model
	Task time variation (A)	The percent of time variation from the preferred basic task time, depending on the skill level of each individual with the tasks in the assigned position.	Task time variation = Training experience + Learning ability + Skill decay	-
Workload	Basic task load** (C)	The personal preference of task load, measured for each individual.	An input constant in REEDCO Score, Newton, seconds, and VACP score, respectively.	-
	Task load** (A)	The task requirement, designed by industrial engineers.	An input constant in REEDCO Score, Newton, seconds, and VACP score, respectively.	-
	Workload** (A)	The perceived difference between basic task load and incoming task load, of each task load component.	Workload = (Task load - Basic task load)/Basic task load + Dynamic effect from circumstantial stressors	-
	Task schedule (C)	The timing of incoming task.	An input sequence of 0 and 1, indicating the status of idle and incoming tasks during the working duration.	-
	Problem occurence (C)	The timing of happening problems, as a sequence of 0 and 1, with 1 indicating the occurrence of problem/failure.	An input sequence * Problem complexity	-
	Working process (A)	A sequence of 0 and 1, representing the schedule of generated incoming tasks and occurring problem/failure.	Task generation = IF THEN ELSE (Task schedule + Problem occurrence = 0 , 0, 1)	-
Personal capacity	Working capacity*(S)	The current level of working capacities of each individual, started from the "Initial personal capacity" in the beginning and degraded throughout the shift.	Personal capacity = Personal initial capacity - Total degradation	-
	Actual task time (A)	The duration that the worker finishes a task.	Actual task time = Basic task time * (100 + Random value of (Task time variation))	-
	Natural degradation*(C)	The capacity degradation rate in a normal working session.	An input constant in percent per minute.	-
	Stressed degradation*(A)	The degradation rate that happens during stressful working duration.	Stressed degradation = Stress effect on capacity degradation	-
	Total degradation*(F)	The total degradation of a working capacity at a certain time	Total degradation = Natural degradation + Stressed degradation	-
	Average situational demand (A)	The average demand from the perceived workload.	Average situational demand = Average of all Workload	-
	Average capability degradation (A)	The average degradation of working capacity.	Average capability degradation = Average of all Working capacity	-
	Perceived situational demand (A)	A sequence of 1 and 0 indicating the status of considering the current task demand exceeds the personal threshold or not, respectively.	Perceived situational demand = IF THEN ELSE (Average situational demand $\geq$ Task demand threshold, 1, 0)	-

Continued on the next page

Type	Variable (#)	Explanation/Reasoning	Value/Equation	Relevant experiment data/theoretical model
**Stress mechanism**	Perceived capability degradation (A)	A sequence of 1 and 0 indicating whether the current capacity degradation exceeds the personal threshold or not, respectively.	Perceived capability degradation = IF THEN ELSE (Average capability degradation $\geq$ Capability threshold, 1, 0)	-
	Perceived demanding task (A)	A sequence of 0 and 1 indicating the status of considering the incoming tasks as not demanding and demanding, respectively.	Perceived demanding task = IF THEN ELSE (Perceived situational demand = 1 :AND: Perceived capability degradation = 1 , 1, 0)	-
	Stress accumulation rate (F)	The accumulation of each type of stress when perceiving the current task is demanding.	Task schedule * Perceived demanding task * stress unit	-
	Stress value (S)	Accumulated values of each type of stress.	Stress value = Stress accumulation rate - Stress relaxation rate	-
	Stress effect on perceived load (A)	The additional load from stress types on perceived workload, negative value (reduced perceived workload) in case of sustained attention, positive value in other cases (increased perceived workload).	Stress effect on perceived load = (Stress value / Stress threshold) * Workload	-
	Stress effect on capacity degradation (A)	The effect from stress, negative value in case of sustained attention, positive value in others.	Stress effect on capacity degradation = (Stress value / Stress threshold) * Natural degradation	-
	Stress relaxation rate (F)	The natural physical relaxation rate from stress value [320], that is in effect while there is no incoming task, or that stress is not accumulating.	Stress relaxation rate = IF THEN ELSE (Task schedule = 0, (Stress value - Stress threshold) / Stress threshold, 0)	-
**Performance profile**	Pace keeping ability (A)	The ability of each individual to keep the "basic work pace" during the working session.	Pace keeping ability = Task time variation + (Psychomotor capacity / Personal initial psychomotor capacity) + Buffer capacity	-
	Distraction occurrence (A)	The probability of distraction or finishing the task late.	Distraction occurrence = Failure rate + (Time task load / Basic task time) + (Work pace / Basic work pace)	-
	Physical reaction (A)	The readiness of physical reaction to perform the expected work movement.	Physical reaction = Posture capacity / Personal initial posture capacity + Force capacity / Personal initial force capacity	-
	Physical degradation (F)	The degradation of physical capability to meet the planned productivity demand.	Physical degradation = Posture stress degradation / Total posture degradation + Force stress degradation / Total force degradation	-
	Attention compensation (A)	The positive effect of Sustained attention on quality performance.	Attention compensation = Stress effect on perceived load (with the value of Sustained attention) + Support readiness	-

Continued on the next page

Type	Variable (#)	Explanation/Reasoning	Value/Equation	Relevant experiment data/theoretical model
	Attention degradation (F)	The degradation of quality-oriented attention.	Attention degradation = Visual stress degradation / Total visual degradation + Cognitive stress degradation / Total cognitive degradation	-
	Availability (A)	The probability that the worker is ready for incoming tasks during the working session.	Availability = Pace keeping ability - Distraction occurrence	-
	Productivity (A)	The probability that the worker can perform the correct task movement in time.	Productivity = Physical reaction - Physical degradation	-
	Quality (A)	The probability that the worker can produce a task output that meets the quality expectation level.	Quality = Attention compensation - Attention degradation	-
	OLE (A)	The personal probability of effective work.	OLE = Availability * Productivity * Quality	-

#: C: Constant / A: Auxiliary / F: Flow / S: Stock
*: posture/force/visual/auditory/cognitive/psychomotor (except ”time”)
**: posture/force/time/visual/auditory/cognitive/psychomotor
stress: sustained attention / acute stress / chronic stress
OLE: Overall Labor Effectiveness
-: Non-applicable
